# Clinical Determinants of Pharmacologic Versus Exercise Stress Testing in Myocardial Perfusion Imaging: A Retrospective Single-Center Study in Saudi Arabia

**DOI:** 10.7759/cureus.114366

**Published:** 2026-08-11

**Authors:** Abdulaziz S Alqahtani, Meshaal M Alenezi, Salem F Alanazi

**Affiliations:** 1 Nuclear Medicine, King Faisal Medical City for Southern Region (KFMCity), Abha, SAU; 2 Nuclear Medicine, King Khalid Hospital (KKHH), Hail Health Cluster, Ministry of Health, Hail, SAU; 3 Radiology, Medical Cities Program, General Directorate of Medical Services, Ministry of Interior, Riyadh, SAU

**Keywords:** coronary artery disease, exercise stress testing, myocardial perfusion imaging, nuclear cardiology, pharmacologic stress testing, saudi arabia

## Abstract

Purpose: Myocardial perfusion imaging (MPI) is a widely utilized noninvasive imaging modality for the evaluation of coronary artery disease and cardiovascular risk stratification. The selection of stress modality during MPI is influenced by multiple demographic and clinical factors. This study aimed to assess the clinical and demographic factors associated with the selection of pharmacologic versus exercise stress testing among patients undergoing MPI at a tertiary care center in Saudi Arabia.

Materials and methods: This retrospective single-center analytical study included adult patients who underwent MPI at a tertiary care hospital in Saudi Arabia. Patients were categorized into exercise stress testing and pharmacologic stress testing groups according to the stress modality utilized during MPI. Demographic and clinical data including age, sex, smoking history, body mass index, cardiovascular risk factors, primary diagnoses, and associated comorbidities were extracted from institutional data collection records. Continuous variables were expressed as mean ± standard deviation, whereas categorical variables were presented as frequencies and percentages. Comparative analyses were performed using independent t-test, chi-square test, and Fisher's exact test where appropriate. Statistical significance was considered at a P-value <0.05.

Results: A total of 197 patients were included in the final analysis. Among them, 147 patients (74.6%) underwent pharmacologic stress testing, whereas 50 patients (25.4%) underwent exercise stress testing. The mean age of the overall study population was 58.8 ± 10.1 years. Patients undergoing pharmacologic stress testing were significantly older than those undergoing exercise stress testing (59.8 ± 10.0 years vs. 55.9 ± 10.2 years, P = 0.02). Significant differences in sex distribution were also observed between the two groups (P = 0.006), with female patients being more frequently represented in the pharmacologic stress group. Patients undergoing pharmacologic stress testing demonstrated a greater prevalence of cardiovascular risk factors, obesity, and exercise-limiting comorbidities. Ischemic heart disease represented the most frequently documented clinical diagnosis within the study population.

Conclusions: Pharmacologic stress testing represented the predominant stress modality among patients undergoing MPI at the tertiary care center. Advanced age, female sex, cardiovascular disease burden, and reduced exercise tolerance were associated with increased utilization of pharmacologic stress testing. Understanding the determinants of stress modality selection may contribute to the optimization of patient-centered imaging strategies, improved workflow efficiency, and enhanced nuclear cardiology practice. These findings may serve as a foundation for the development of a standardized clinical protocol to guide stress modality selection in MPI. Implementation of evidence-based selection criteria may improve consistency in clinical decision-making, optimize patient safety, and enhance the efficiency of nuclear cardiology services.

## Introduction

Myocardial perfusion imaging (MPI) is one of the most widely used noninvasive diagnostic procedures in modern-day nuclear cardiology for the assessment of coronary artery disease (CAD). It is important for the evaluation of myocardial ischemia, cardiac risk stratification, myocardial viability assessment, and treatment decision-making in patients with suspected or confirmed cardiovascular disease [1]. Single-photon emission computed tomography (SPECT) MPI remains the most commonly performed nuclear cardiology procedure worldwide due to its proven diagnostic accuracy, prognostic value, and clinical utility in both symptomatic and asymptomatic patients at risk of CAD [2].

Stress testing is an integral component of MPI to assess myocardial blood flow under augmented cardiac demand. The diagnostic ability of MPI relies mainly on how well stress induction is performed [3]. The two main types of stress used in clinical practice are exercise stress testing and pharmacologic stress testing [4,5]. Exercise stress testing is generally preferred when feasible because it provides additional physiologic and prognostic information such as exercise tolerance, functional capacity, hemodynamic response, blood pressure change, heart rate recovery, and exercise electrocardiographic abnormalities [5,6]. However, many patients referred for MPI cannot undertake acceptable levels of physical activity because of old age, obesity, musculoskeletal illness, peripheral vascular disease, pulmonary disease, cognitive impairment, or severe cardiovascular restrictions [7]. For perfusion measurement, pharmacologic stress testing is a reliable option in such individuals to generate coronary vasodilation or increased myocardial oxygen demand [8,9]. Pharmacologic stress medications often used include adenosine, dipyridamole, regadenoson, and dobutamine, each having unique indications and contraindications [5,10].

The choice of stress modality is based on a number of demographic, clinical, and operational considerations [11-13]. Factors affecting the decision-making process for stress modality selection may include, but are not limited to, age, sex, body mass index (BMI), diabetes mellitus, hypertension, smoking history, respiratory illness, functional status, and baseline cardiovascular risk [12]. Institutional rules, physician choice, patient safety concerns, and logistical reasons may also contribute to variations in clinical practice patterns [4,5].

Several studies have examined factors associated with the use of pharmacologic versus exercise stress testing. Previous findings have shown that the increasing use of pharmacologic stress testing is associated with older age, female sex, obesity, lower exercise tolerance, diabetes mellitus, hypertension, chronic obstructive pulmonary disease, and established cardiovascular disease [14,15]. However, patterns of stress modality usage may vary widely across institutions and geographic areas, reflecting variations in patient demographics, referral procedures, healthcare systems, and institutional resources [5,16]. Cardiovascular disease is one of the leading causes of morbidity and mortality in Saudi Arabia. The increasing prevalence of diabetes mellitus, hypertension, obesity, and sedentary lifestyle has increased the demand for MPI [14,15]. Nuclear cardiology services are becoming increasingly utilized in Saudi Arabia [17,18]. However, there is a paucity of published data on the factors influencing the choice of stress modality among patients receiving MPI in Saudi Arabia [19]. Understanding local factors influencing stress modality selection may help optimize patient care and support evidence-based nuclear cardiology practice [12,20]. Therefore, this study aims to assess the clinical and demographic factors influencing the selection of pharmacologic versus exercise stress testing among adult patients undergoing MPI at a tertiary care hospital in Saudi Arabia [7,21].

## Materials and methods

Study design and setting

The research was a retrospective analytical study at a single site performed in a tertiary care hospital in Saudi Arabia. Data were retrospectively collected from adult patients who underwent MPI during the period from January 2022 to December 2025. The research employed institutional records and data collection sheets from adult patients undergoing MPI for regular clinical assessment of suspected or confirmed cardiovascular disease [17,18]. A retrospective analytical approach was used in this research to assess demographic and clinical factors associated with stress modality selection in MPI as previously published. The research was done in a tertiary care facility, a major referral center for nuclear cardiology services, and serves patients from many parts of the nation [10]. The hospital regularly performs MPI according to defined nuclear medicine procedures and under the supervision of competent nuclear medicine specialists and cardiologists. Exercise and pharmacologic stress tests are both routinely performed based on clinical state, exercise tolerance, and clinician judgment. The retrospective approach enabled assessment of real-world clinical practice patterns and factors related to stress modality use in normal nuclear cardiology practice [1,12].

Ethics approval

This retrospective study was approved by the Institutional Review Board (IRB) of Jeddah Health Affairs, Ministry of Health, Saudi Arabia (IRB Approval No. A02257; approval date: 9 July 2026). The study was conducted using retrospectively collected anonymized patient data obtained from institutional records. All study procedures were conducted in accordance with the ethical principles of the Declaration of Helsinki. 

Study population

The study population consisted of all eligible adult patients who underwent MPI between January 2022 and December 2025. Patients were included regardless of sex or cardiovascular diagnosis if comprehensive demographic and clinical information were available in the institutional records. Patients were divided into two groups based on the modality of stress utilized for MPI: exercise stress testing group and pharmacologic stress testing group [17,19]. Exercise stress testing was typically conducted in patients who could achieve appropriate physical effort using treadmill-based stress regimens in accordance with institutional norms. Pharmacologic stress testing was used in individuals who were unable to exercise satisfactorily because of old age, obesity, cardiovascular restrictions, respiratory illness, musculoskeletal diseases, neurological impairment, or other clinical contraindications to exercise stress testing [18,22]. The exclusion criteria were inadequate clinical data, lack of documentation of the stress modality, and incomplete or conflicting medical records. Duplicate records and missing data entries were also removed to guarantee consistency and quality of statistical analysis [13].

Data collection

Data were retrieved retrospectively from institutional data collection sheets and patient records using a standardized data extraction technique. Patient age and sex were collected as demographic information. Clinical and anthropometric characteristics included weight, height, smoking status, cardiovascular risk factors, main diagnosis, and associated medical comorbidities. BMI was calculated from weight and height data using the standard formula of weight in kilograms divided by height in meters squared. Smoking history was obtained if possible, and was classified according to documented smoking status [2,14]. Clinical variables related to cardiovascular risk factors and comorbidities were also collected including hypertension, diabetes mellitus, obesity, ischemic heart disease, respiratory disease and other relevant chronic medical conditions that could potentially affect the choice of stress modality [10,23]. Furthermore, the main justifications for MPI were examined and included the assessment of chest pain, suspected CAD, assessment of myocardial ischemia, aberrant electrocardiographic findings, and evaluation of cardiovascular symptoms in high-risk individuals [24]. To eliminate information bias and increase the reliability of the data, all data were checked for completeness and consistency before inclusion in the statistical analysis [17,25].

Stress modality assessment

The main outcome variable of the research was the choice of stress modality for MPI. Stress modality was characterized as exercise stress test or pharmacologic stress test. Exercise stress testing was conducted using treadmill exercise protocols in accordance with institutional nuclear cardiology norms. Patients were urged to reach target heart rate values when clinically possible for better diagnostic accuracy and prognostic prognosis [8,20]. Exercise stress testing is usually recommended as a stress modality because it gives extra physiologic and functional information including exercise tolerance, blood pressure response, heart rate recovery, and exercise-induced electrocardiographic alterations [3]. However, pharmacologic stress testing was done in individuals who could not exercise adequately due to physical restrictions or underlying medical disorders. Standard pharmacologic stress agents were used for pharmacologic stress protocols according to institutional nuclear medicine standards and patient-specific contraindications. The choice of stress modality was made by the supervising physician after thorough examination of the patient's clinical state, functional ability, cardiovascular condition, and procedural safety factors. This study aimed to assess the demographic and clinical factors associated with the selection of pharmacologic versus exercise stress testing in routine clinical practice [4,5].

Statistical analysis

All statistical analyses were performed using IBM SPSS Statistics for Windows, Version 27.0 (Released 2019; IBM Corp., Armonk, New York, USA). Data cleaning and verification processes were carried out prior to statistical analysis to detect missing values, duplicate entries, and inconsistent data. Continuous variables were reported as mean ± SD and categorical variables as frequency and percentage. Descriptive statistical analysis was conducted to summarize demographic parameters, clinical factors, and the distribution of stress modality in the study population [16,18]. Comparative studies were then conducted comparing individuals receiving exercise stress testing to those undergoing pharmacologic stress testing. Continuous variables were compared using the independent-samples t-test when normally distributed or using appropriate nonparametric test when the assumption of normality was not met. Categorical variables were compared using the chi-square test or Fisher's exact tests according to the expected cell frequencies [3,21]. Variables considered for comparison study were age, gender, smoking history, BMI, diabetes mellitus, hypertension, obesity, cardiovascular disease, and associated medical comorbidities. Binary logistic regression analysis was designed to discover independent factors linked with pharmacologic stress testing [19,22]. The dependent variable in the regression model was stress modality, whereas the independent variables were demographic and clinical features. Odds ratios with matching 95% confidence intervals were developed to measure the strength of connection between clinical factors and the use of pharmacologic stress testing. A P-value <0.05 was considered statistically significant for all statistical analyses [3,7].

## Results

This study showed that pharmacologic stress testing was the most commonly used stress modality in patients who underwent MPI at the tertiary care facility. Of the total study population of 197 patients, 147 patients (74.6%) had pharmacologic stress testing and 50 patients (25.4%) had exercise stress testing. There were notable demographic variations between exercise and pharmacologic stress groups, especially with regard to age and female distribution. Patients receiving pharmacologic stress testing were typically older (mean age, 59.8 ± 10.0 years) than those in the exercise stress group (mean age, 55.9 ± 10.2 years). Increasing age was significantly associated with the use of pharmacologic stress testing (independent-samples t-test, P = 0.02). Moreover, female patients were more represented in the pharmacologic stress group, whereas male patients were predominant in the exercise stress group, showing a statistically significant difference in sex distribution across both modalities (chi-square test, P = 0.006).

Further review of clinical records showed that patients referred for pharmacologic stress testing have a higher frequency of cardiovascular risk factors, obesity, lower functional capacity, and associated exercise-limiting comorbidities. The most prevalent recorded diagnosis in the study population was ischemic heart disease followed by dyslipidemia, obesity, hypertension, thyroid disorders, arrhythmia, and chronic renal disease. Patients with several chronic medical illnesses and decreased exercise tolerance were more likely to need pharmacologic stress treatments to achieve sufficient myocardial stress induction. However, exercise stress testing was used more often in younger individuals with retained functional ability and less indicated clinical restrictions. These patients were typically able to reach goal activity levels in treadmill stress protocols and gained extra functional and prognostic information from exercise testing. The results of this research emphasize the substantial effect of demographic and clinical variables on stress modality selection in nuclear cardiology practice. Enhanced knowledge of these variables may lead to improvement of patient-centered imaging procedures, increased procedural safety, better patient selection, and more effective workflow management in nuclear medicine departments.

Study population and stress modality distribution

In the final analysis, the total number of adult patients who underwent MPI throughout the research period was 197. Patients were stratified into exercise stress testing and pharmacologic stress testing groups based on the stress modality used during MPI. Of the enrolled patients, 50 (25.4%) had exercise stress testing and 147 (74.6%) had pharmacologic stress testing. The high rate of pharmacologic stress testing in this study is a consequence of the high prevalence of cardiovascular risk factors and exercise-limiting clinical conditions among patients referred for MPI at our tertiary care facility. Exercise stress tests were usually done on patients who could exercise adequately and who had intact physical ability, while pharmacologic stress tests were used more often in patients who were older, had many comorbid conditions, or had impaired functional ability (Table 1).

**Table 1:** Distribution of stress modality among study population.

Variables	Frequency (n)	Percentage
Exercise stress testing	50	25.4
Pharmacologic stress testing	147	74.6
Total study population	197	100

Demographic characteristics

Demographic characteristics of the study population are reported in Table 2. The entire study population was mostly male patients; however, female patients made up a large percentage of patients who underwent pharmacologic stress testing. The average age of the study population was around 58.8 ± 10.1 years. Patients who underwent pharmacologic stress tests were older than those who underwent exercise stress tests. The mean age of the exercise stress group was 55.9 ± 10.2 years, whereas the mean age of the pharmacologic stress group was 59.8 ± 10.0 years. The independent-samples t-test showed a statistically significant difference in age between the two groups, *t*(195) = -2.37, P = 0.02. Analysis of sex distribution also revealed statistically significant variations between the two stress modality groups. Male patients were 64.0% in the exercise stress group and 40.1% in the pharmacologic stress group. Among patients undergoing pharmacologic stress testing, female patients were more commonly represented. Sex distribution across groups was significantly different (chi-square test, P = 0.006).

**Table 2:** Baseline demographic characteristics of patients undergoing MPI. Continuous variables were compared using the independent-samples t-test. Categorical variables were compared using the chi-square test. MPI: myocardial perfusion imaging.

Variables	Exercise stress (n = 50)	Pharmacologic stress (n = 147)	Total (n = 197)	P-value
Age (years), mean ± SD	55.9 ± 10.2	59.8 ± 10.0	58.8 ± 10.1	0.02
Male sex, n (%)	32 (64.0)	59 (40.1)	91 (46.2)	0.006
Female sex, n (%)	18 (36.0)	88 (59.9)	106 (53.8)	0.006

Clinical characteristics and comorbidities

Review of clinical records showed considerable diversity in cardiovascular risk factors and related medical comorbidities across the enrolled individuals. Several patients had various chronic medical illnesses, consistent with the complicated cardiovascular risk profile often seen in a tertiary care nuclear cardiology practice. The clinical diagnoses most often reported were ischemic heart disease, dyslipidemia, obesity, hypertension, diabetes mellitus, respiratory illness, arrhythmia, chronic kidney disease, and thyroid disorders. Patients undergoing pharmacologic stress testing generally had a greater burden of cardiovascular risk factors and exercise-limiting conditions than those undergoing exercise stress testing. The most prevalent main diagnosis recorded was ischemic heart disease. Other reasons for MPI included assessment of chest discomfort, aberrant electrocardiographic findings, dyspnea on exercise, and cardiovascular risk assessment in high-risk individuals. Patients with advanced cardiovascular illness, obesity, pulmonary disease, musculoskeletal restrictions, and poor functional status were more likely to undergo pharmacologic stress testing due to difficulties to safely and efficiently attain appropriate exercise-induced cardiac stress levels (Table 3).

**Table 3:** Frequently documented clinical diagnoses and comorbidities.

Clinical variables	Frequency (n)
Ischemic heart disease	12
Dyslipidemia	5
Obesity	2
Thyroid disorders	2
Dyspnea	1
Arrhythmia	1
Hypertension	1
Chronic kidney disease	1
Other associated comorbidities	Multiple

Age-based comparison between stress modalities

Age-based analysis indicated a positive correlation between patient age and the use of pharmacologic stress testing. Older patients usually had lower exercise tolerance due to cardiovascular disease, obesity, respiratory restrictions, overall physical deconditioning, or musculoskeletal disorders. Therefore, pharmacologic stress testing was more often used in older patients to induce a sufficient and safe cardiac stress during imaging. Exercise stress testing was performed more often in younger patients, who had retained functional ability and fewer contraindications to treadmill exercise regimes. The exercise stress test included predictive information on exercise tolerance, hemodynamic response, and functional cardiovascular performance. The statistically significant difference in age between the exercise and pharmacologic stress groups implies that age is an important factor in the choice of the stress modality used during MPI.

Sex-based analysis

When analyzed by sex, there were significant disparities in the use of stress modalities between male and female patients. Male patients more frequently underwent exercise stress testing, whereas female patients more frequently underwent pharmacologic stress testing. The observed disparity may be due to many causes including differences in exercise tolerance, obesity prevalence, musculoskeletal restrictions, cardiovascular risk load, and related chronic medical disorders. In routine nuclear cardiology practice, female patients often have lower exercise capacity and may therefore require pharmacologic stress protocols more often. The findings of this study are largely in agreement with the previously published nuclear cardiology literature documenting increased use of pharmacologic stress testing among female patients and elderly populations.

Comparative statistical analysis

Statistical analysis of the comparative nature showed substantial variations in demographic factors between the exercise stress and the pharmacologic stress groups. The independent t-test analysis showed statistically significant differences in the patient age between groups (P = 0.02). Similarly, chi-square analysis indicated statistically significant differences in sex distribution (P = 0.006). The results show that demographic variables are clinically significant determinants of stress modality selection for MPI. Older age and female sex were related with a larger incidence of pharmacologic stress testing. Due to the diversity in recording among institutional records, formal statistical examination of all comorbidities was restricted. However, descriptive analysis indicated a definite trend toward a greater cardiovascular risk burden among patients undergoing pharmacologic stress testing (Table 4).

**Table 4:** Comparison of demographic characteristics between exercise and pharmacologic stress groups. Continuous variables were compared using the independent-samples t-test, and categorical variables were compared using the chi-square test. The comparison of age between the exercise and pharmacologic stress groups yielded  *t*(195) = -2.37, P = 0.02.

Variables	Exercise stress	Pharmacologic stress	Statistical test	P-value
Mean age	55.9 ± 10.2 years	59.8 ± 10.0 years	Independent t-test	0.02
Male sex	64.0%	40.1%	Chi-square test	0.006
Female sex	36.0%	59.9%	Chi-square test	0.006

Predictors associated with pharmacologic stress testing

Descriptive analysis of the study population showed that a number of demographic and clinical characteristics were related with greater usage of pharmacologic stress testing. One of the biggest factors for choice of pharmacologic stress treatment that we identified was advanced age. Similar trends were shown for females and the existence of activity limiting comorbidities, which tend to enhance the risk of using pharmacologic stress. Pharmacologic stress testing was often necessary in patients with obesity, cardiovascular disease, pulmonary disease, musculoskeletal disorders, or broad physical limitations since treadmill exercise programs were unable to reach appropriate target heart rate values. We intended to use binary logistic regression analysis to identify independent factors related with pharmacologic stress testing. Regression analysis was carried out including age, sex, smoking status, hypertension, diabetes mellitus, obesity, cardiovascular disease, and related chronic medical diseases. The goal of multivariable analysis was to identify clinical parameters independently associated with pharmacologic stress testing after accounting for possible confounding variables.

## Discussion

This study examined the clinical and demographic parameters influencing the decision of pharmacologic versus exercise stress testing in patients undergoing MPI in a tertiary care hospital in Saudi Arabia. Results suggested that pharmacologic stress testing was the most frequent stress modality used in the study population, comprising about three-quarters of all MPI procedures. In addition, there were significant differences in the age and sex distribution between exercise and pharmacologic stress groups, which underscore the importance of patient demographics and related clinical characteristics in the selection of stress modality in routine nuclear cardiology practice [3]. It was shown that the mean age of patients undergoing pharmacologic stress testing was significantly higher than that of patients undergoing exercise stress testing. Older persons may have poorer exercise tolerance due to cardiovascular disease, musculoskeletal limitations, obesity, pulmonary disease, generalized physical deconditioning, and other chronic medical conditions. This indicates that pharmacologic stress testing is necessary in many elderly patients who are unable to achieve adequate target heart rate values during treadmill exercise regimens. These findings are consistent with recent studies demonstrating that older age is one of the strongest predictors of increased pharmacologic stress testing during MPI [15,17,26].

This study also identified significant differences in sex distribution between the two sets of stress modalities. Exercise stress testing was more frequent among males, while women accounted for a larger proportion of the pharmacologic stress group. There are several possible explanations for this discovery. Female patients usually have poorer exercise tolerance and a greater prevalence of obesity, musculoskeletal problems, and chronic medical conditions that limit activity than male patients [10,18,22]. Limitations associated to anxiety and inferior baseline functional ability may also contribute to increased utilization of pharmacologic stress regimens in women. Similar findings have been reported in previously published nuclear cardiology literature, showing repeatability of the present results.

The other significant result of this study was the greater prevalence of cardiovascular risk factors and associated comorbidities in patients referred for pharmacologic stress testing. The review of clinical records showed that ischemic heart disease, hypertension, diabetes mellitus, dyslipidemia, obesity, pulmonary disease, thyroid disorders, and chronic kidney disease are often present together in patients referred for pharmacologic stress tests. Pharmacologic stress induction is often needed for myocardial perfusion assessment in individuals with different chronic medical issues who cannot safely and efficiently do adequate physical exercise [2,12,23].

The most common clinical diagnosis in the study population was ischemic heart disease. This finding is expected clinically since MPI is primarily used for the assessment of suspected or established CAD and for the qualitative and semi-quantitative assessment of myocardial ischemic burden. The growing demand of MPI services in tertiary care nuclear medicine departments is probably due to the rising prevalence of cardiovascular disease and associated metabolic risk factors in Saudi Arabia. The rising burden of diabetes mellitus, hypertension, obesity, and sedentary lifestyle in the Saudi population, in particular, has greatly increased the number of patients who require advanced cardiovascular imaging and functional cardiac evaluation [8,12,15]. The predominance of pharmacologic stress testing in this study may well mirror referral patterns often observed in tertiary healthcare settings. Patients sent to tertiary care centers are frequently high-risk patients with severe cardiovascular disease and numerous other comorbidities. Pharmacologic stress testing is often the main or only feasible way to induce myocardial stress in these people, who frequently have limited functional capacity and low exercise tolerance [12,16,23].

On the other hand, exercise stress protocols are often better suited for younger patients with less clinical limitations. However, exercise stress testing remains therapeutically relevant, providing important functional and prognostic information beyond the perfusion imaging findings. Further cardiovascular risk assessment is based on the predictive significance of the exercise-generated electrocardiographic abnormalities, blood pressure response, exercise capacity, chronotropic competence, and heart rate recovery. Thus, exercise stress testing continues to be the preferred stress modality in nuclear cardiology practice when clinically feasible. However, pharmacologic stress testing has an essential adjunctive role in patients who are unable to attain satisfactory levels of exercise or have contraindications to exercise programs [5,12,26]. The findings of this study are generally consistent with previously published international research evaluating parameters of stress modality choice in MPI. There is a strong association between the use of pharmacologic stress and older age, female sex, obesity, diabetes mellitus, hypertension, respiratory sickness, and reduced exercise capacity, as shown in several studies. This study offers useful regional data from Saudi Arabia on the patterns of stress modality use and clinical aspects of patients evaluated with MPI in routine tertiary care environment [2,16].

Clinically, awareness of the factors associated with the selection of a certain stress modality may foster patient-centered approaches to imaging and aid in optimizing workflow management within nuclear cardiology. Appropriate patient selection may enhance the safety of the process, reduce unnecessary testing delays, improve imaging quality, and allow for more efficient use of institutional resources. Additionally, identifying patients who are likely to undergo pharmacologic stress testing prior to scheduling such testing might improve operational planning in nuclear medicine departments and decrease procedural inefficiencies [19]. This study has several strengths. First, it provides genuine clinical data about the use of stress modalities in a tertiary care setting in Saudi Arabia where there is currently a lack of regional data. Second, the study examined a broad range of demographic and clinical factors associated with the choice of stress modality. Third, by including both exercise and pharmacologic stress groups, we were able to directly compare patient characteristics across modalities [10,21].

Some limitations should be mentioned, however. The retrospective and single-center nature of this study may limit generalizability to other institutions or healthcare systems. As the study was based on institutional data, several clinical variables may be incompletely recorded, thereby affecting the completeness of the statistical analysis. Variability in recording across patient records further limited the quantitative assessment of all cardiovascular comorbidities and exercise-limiting illnesses. Additionally, the retrospective design may have introduced selection and information bias because the study relied on routinely collected clinical records rather than prospectively collected data collection. The lower number of patients in the exercise stress group compared with the pharmacologic stress group may have also influenced the comparative statistical interpretation [6,15]. Additionally, the relatively modest overall sample size may have limited the statistical power and generalizability of the findings. However, the study included all eligible patients who underwent MPI at our institution between January 2022 and December 2025, representing the complete retrospective dataset available at our institution during the study period. Larger prospective, multicenter studies with a standard clinical documentation and larger sample size are needed to properly investigate variables connected with stress modality selection during MPI. Further study on the evaluation of the procedure findings, diagnostic accuracy, patient safety, and long-term prognostic implications of the use of stress modalities would further strengthen the evidence-based nuclear cardiology practice in the field.

In summary, the findings of this study demonstrate a strong influence of demographic and clinical parameters on the selection of stress modality in MPI. Pharmacologic stress testing is more common in the elderly and in people with multiple cardiovascular risk factors or activity restricting medical issues. Greater awareness of these features may facilitate the optimization of patient-centered nuclear cardiology services and improve the efficiency of the diagnostic process in tertiary healthcare institutions [9,22].

## Conclusions

Pharmacologic stress testing was the predominant stress modality among patients undergoing MPI at our tertiary care center, accounting for approximately three-quarters of all examinations. Significant differences were observed between the exercise and pharmacologic stress groups, with pharmacologic stress testing being more frequently selected for older patients, women, and those with a greater burden of cardiovascular risk factors, chronic medical conditions, and exercise-limiting comorbidities. These findings suggest that clinical and demographic characteristics play an important role in stress modality selection and may support the future development of standardized, evidence-based protocols. Further multicenter studies with larger sample sizes are warranted to validate these findings.
